# Smartphone applications for the management of epilepsy in children and adolescents

**DOI:** 10.1590/1984-0462/2025/43/2024066

**Published:** 2024-09-06

**Authors:** Gabriela Garcia de Carvalho Laguna, Diego Bastos Ribeiro, Lidhane Santos Coelho, Murilo Sousa Ramos, Karolaine da Costa Evangelista, David Santos Libarino, Davi Tanajura Costa

**Affiliations:** a Universidade Federal da Bahia, Instituto Multidisciplinar em Saúde – Vitória da Conquista, BA, Brazil.; b Universidade Federal do Sul da Bahia – Teixeira de Freitas, BA, Brazil.; c Universidade Estadual do Sudoeste da Bahia – Vitória da Conquista, BA, Brazil.

**Keywords:** Epilepsy, Self-care, Gamification, Child, Adolescent, Epilepsia, Autocuidado, Gamificação, Criança, Adolescente

## Abstract

**Objective:**

To describe how smartphone applications can contribute to the management of epilepsy in children and adolescents.

**Data source:**

This is an integrative review conducted on the Medline, PubMed, and SciELO databases, based on the descriptors “epilepsy” and “smartphone.” Original studies published between 2017–2023 in Portuguese or English that addressed the research question were included. Theses and dissertations, duplicate studies, literature reviews, and studies that did not answer the research question were excluded.

**Data synthesis:**

A total of 178 studies were located, of which six were selected for this review. The sample included 731 participants (631 children and adolescents with epilepsy and 100 caregivers). The applications allow for the collection of seizure frequency; timing and type of crisis; reminders for medication administration; and information about sleep quality. They can store these data for healthcare professionals, caregivers, and users to monitor the progress of the condition.

**Conclusions:**

The use of applications in managing seizures in children and adolescents with epilepsy shows promising results by promoting continuous and personalized monitoring. Further studies are needed to optimize beneficial outcomes and overcome challenges.

## RESUMO

**Objetivo:**

Descrever como aplicativos de smartphone podem contribuir para o gerenciamento de quadros de epilepsia em crianças e adolescentes.

**Fontes de dados:**

Trata-se de uma revisão integrativa realizada nas bases de dados Medline; PubMed e SciELO, com base nos descritores “*epilepsy*” e “*smartphone*”. Foram incluídos estudos originais publicados entre 2017–2023 em português ou inglês que respondessem à pergunta de investigação. Teses e dissertações, estudos duplicados, revisões de literatura e estudos que não responderam à questão de pesquisa foram excluídos.

**Síntese dos dados:**

Foram localizados 178 estudos, dos quais seis foram selecionados para compor esta revisão. A amostra incluiu 731 participantes (631 infantojuvenis com epilepsia e 100 cuidadores). Os aplicativos permitem a coleta da frequência de convulsões; momento e o tipo de crise; lembretes para a administração de medicamentos; e informações sobre a qualidade do sono, sendo capazes de armazenar esses dados para profissionais da saúde, cuidadores e usuários acompanharem a evolução do quadro.

**Conclusões:**

O uso de aplicativos no manejo das convulsões de crianças e adolescentes com epilepsia apresenta resultados promissores ao promoverem um monitoramento contínuo e personalizado. Novos estudos são necessários para otimizar os resultados benéficos e superar desafios.

## INTRODUCTION

Epilepsy is a neurological disorder that affects approximately 50 million people worldwide. It is characterized by seizures that occur either two or more times, unprovoked, more than 24 hours apart, or at least once when the person has at least a 60% chance of having new episodes within ten years. In cases of epileptic syndrome, specific findings are used to diagnose the condition.^1,2,3^

The disease is a chronic condition that requires multiple approaches for effective treatment, in addition to medication. People with epilepsy need to make various psychosocial and behavioral adaptations to their lifestyle habits to control the condition and prevent risks. Regular sleep, stress control, exercise, and avoiding alcohol and tobacco are all essential components of self-management for epilepsy.^4^ Effective epilepsy control requires collective efforts from the healthcare team, patients, and caregivers. This can be achieved by ensuring medication adherence, monitoring symptoms, and adopting healthy habits.

This is one of the most common neurological conditions in children, often associated with comorbidities such as intellectual disability, learning disabilities, depression and anxiety — both conditions related to stress, which can either trigger seizures or be a result of them —, attention deficit hyperactivity disorder, sleep disorders, and bone problems.^2,5^ Family, caregiver, and child/adolescent should be guided on disease management, which includes safety measures for seizures, first aid, and medications; it is also important to establish and annually update a seizure action plan, ensuring that those in the environments frequented by the person are aware of it.^2^ Therefore, the significant psychosocial repercussions related to the condition are undeniable.

Advancements in technology have allowed for the development of smartphone apps that cater to self-care and management of chronic conditions such as epilepsy, some of which are targeted towards young people and children.^6^ Since the COVID-19 pandemic, there has been an increase in studies on the effectiveness of smartphone apps in monitoring epilepsy in children and adolescents. However, the existing literature lacks systematic reviews on this subject. Therefore, this research aimed to describe how smartphone apps can contribute to the management of epilepsy in children and adolescents.

## METHOD

This is an integrative literature review that had the purpose of synthesizing knowledge and facilitating its practical application. The review follows six stages as outlined by Botelho et al.:^7^

1) Identify the topic and select the research question;
2) Establish inclusion and exclusion criteria;
3) Identify the pre-selected and selected studies;
4) Categorize the selected studies;
5) Analyze and interpret the results; and
6) Present the review or synthesis of knowledge.

This review aimed to investigate the role of smartphone apps in managing epilepsy in children and adolescents and assess their effectiveness. The article search was conducted in March 2023, using the Medical Literature Analysis and Retrieval System Online (Medline), PubMed, and Scientific Electronic Library Online (SciELO) databases. The search terms included “epilepsy” and “smartphone”, along with their synonyms, combined, and using the Boolean operators “AND” and “OR”.

This study includes original research published in English and Portuguese between 2017 and 2023. The publications should cover the use of smartphone apps in the management of epilepsy and be relevant to children and young adults up to 18 years old. Theses and dissertations, duplicate studies, literature reviews, and studies that did not answer the research question were not included.

Data were recorded in an Excel spreadsheet, analyzed, and then displayed in a table (Table 1)^8,9,10,11,12,13^ taking into account the analyzed variables such as author, year, location, study design, sample, tool used, and main results. The Rayyan web application was employed to facilitate the organization of the included studies.^14^

**Table 1:** Characterization of the studies and main findings.

Study	Study design and location	Sample	Tool used	Main results and conclusions
Chiu et al.^8^	Cross-sectional observational (Canada)	192 participants: average age 10.4 years.	This study aimed to determine the percentage of pediatric patients with epilepsy who have individualized action plans, identify predictive variables, and assess parental interest in a mobile application with SAP guidelines.	The study showed that 15% of parents use mobile apps to manage their children’s epilepsy, with the most commonly used features being seizure trackers and medication reminders, and that 83% of participants are interested in an app that presents an individualized action plan for seizures.
Choi et al.^9^	Longitudinal observational (South Korea)	99 participants: 18 patients aged 15 years or over and 81 caregivers.	Brain4U mobile application, which includes a seizure diary, medication reminders, comorbidity self-screening tools, and an individualized dashboard for epilepsy patients.	The app provided accurate information on the timing and type of seizures but had variations in user adherence and some inconsistencies in clinical data. Caregivers and patients reported that educational content and medication reminders were the most useful features.
Davies et al.^10^	Observational (South Africa)	39 participants: average age 10 years (4–6 years)	Smartphone application (Aparito app) and pulse device connected via Bluetooth.	The app can capture important information such as medication adherence data and clinical events. Crisis management becomes more effective.
Dozières-Puyravel et al.^11^	Cross-sectional observational (France)	36 participants: 17 adolescents aged 10–18 years and 19 parents or caregivers.	A questionnaire for teenagers to understand their interests and the type of content they would like to see in an epilepsy self-management app.	Most parents and adolescents were interested in an epilepsy self-management app. A lack of knowledge about existing apps was observed and it is believed that the use of these tools could promote greater dissemination of knowledge about epilepsy.
LaGrant et al.^12^	Observational (the United States)	314 pediatric participants with average onset of symptoms of 2.8.	Electronic seizure diary that allows the entry of demographic information and recording of seizures via mobile devices, wearable devices, or Amazon Alexa.	The study evaluated spasm patterns in epileptic children using an electronic diary available on mobile devices and observed individual differences between users of the electronic diary in terms of the frequency of reports.
Le Marne et al.^13^	Observational (Australia)	51 participants: average age 14.49 years.	The EpApp application for epilepsy management. It uses medication reminders, a seizure diary, and personalized statistics.	During the study, there was a significant improvement in general knowledge about epilepsy, but not in attitudes or self-efficacy related to the disease. Adolescents and parents considered medication reminders to be the most useful resource.

## RESULTS

**Figure 1:**
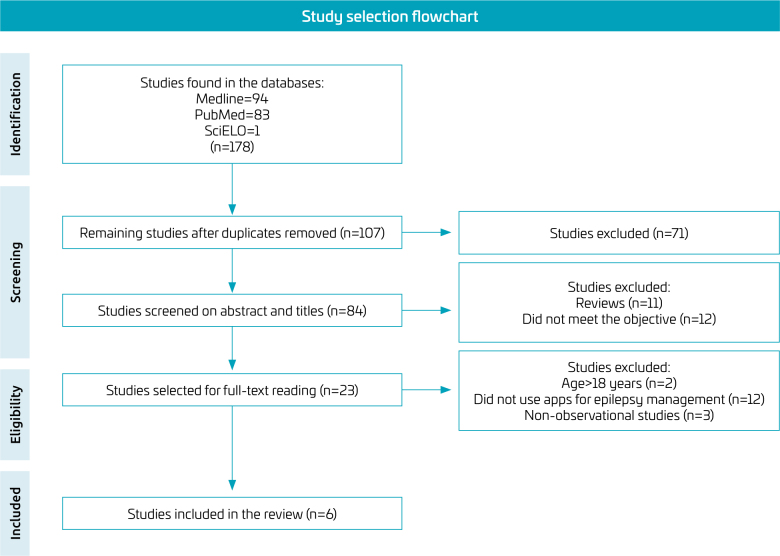
Study selection flowchart.

The literature search identified 178 articles, six of which were selected to make up the sample for this review, as shown in Figure 1 (study selection flowchart).

This review comprised six studies, one longitudinal and five cross-sectional, published between 2018 and 2021. They included a sample of 731 participants, of which 631 were pediatric patients (aged between 0–18 years) diagnosed with epilepsy, and the remaining 100 were caregivers of patients with this diagnosis. The studies were conducted in different countries including South Africa, the USA, Canada, France, South Korea, and Australia.

Two of them identified the type of epilepsy in their sample, detecting tonic-clonic seizures, absence seizures, focal epilepsy, refractory epilepsy, and epilepsy associated with cognitive deficits.^10,11,12,13^ The other studies did not specify the type of epilepsy of the participants. In all articles, participants were recruited through health centers and referral hospitals for epilepsy treatment.

Most of the devices described consisted of self-management via apps, with seizure diaries, reminders for medication, and scheduled appointments, data collection for seizure tracking, and the provision of informative content on care for epileptic patients.^9,10,13^ In one of the articles, the app was integrated into the patient’s electronic hospital records, making it possible to record more specific information about the seizures such as dates, types, number in each type, duration, and when they occurred, as well as brief descriptions.^9^

The use of external devices was reported in only one of the selected studies; it was a wrist device integrated with an app via Bluetooth connection, capable of recording steps, heart rate, duration and type of sleep, in addition to the other features already mentioned. The authors revealed that the use of the app to record seizures was more frequent than the paper seizure diary; therefore, they concluded that apps are advantageous, as well as more appropriate for therapeutic management.^10^

The presence of parents and caregivers in the monitoring of epilepsy was observed in five of the selected studies; these agents assisted the participants in recording, as well as in observing changes in behavior.^8,9,10,13^

The analysis of the improvement in medication adherence was addressed in one study, which compared the need for medication reminders before and during the period of the app usage; the authors reported that fewer reminders were needed throughout the intervention.^13^ Other authors presented feedback from parents, caregivers, and patients on the usefulness of the tools. Of these users, 70% were positively satisfied with the management of crises.^8^ Parents and caregivers mentioned educational content and medication reminders as the most useful resources, while adolescents highlighted only reminders as the most useful tool.^8,13^

## DISCUSSION

In light of the desire for autonomy in self-managing epileptic seizures, there has been, in recent years, an increase in the development of apps that assist epilepsy patients and their caregivers in managing and understanding these situations through technological tools with various functionalities that can be accessed via electronic devices such as smartphones or tablets. The need to optimize seizure control stems from the importance of minimizing the impacts of this chronic disease to promote a better quality of life. These daily implementations of specific strategies tend to have a greater impact on children and adolescent, due to a vulnerability stemming from emotional immaturity that predisposes them to psychopathologies and alterations in physical, cognitive, and psychosocial development.^15^

A series of studies involving adolescents aged 10–18 years, parents, and caregivers were conducted, jointly between multidisciplinary teams and patients, aiming to gather data on knowledge about smartphone apps for epilepsy management, interest in using these mobile tools, and expectations associated with their use. Among the desired functionalities, most parents demonstrated interest in a mobile application that could record the number and duration of daily seizures, while adolescents preferred reminders related to the timing of medication intake. Both groups found it relevant to have a list of contraindicated medications, instructions for treatment and emergency, a medical appointment planner, and the possibility of easy access to the physician responsible. Information on sports practices, professions, and data regarding different anticonvulsant medications were also pointed out as necessary utilities. The individualization of actions carried out through seizure action plans for the pediatric population with epilepsy that could be included in the apps constituted another facet of this reality, which represents an attempt to equip these individuals with the necessary knowledge for seizure management. From this perspective, there was a strong interest in a mobile application that could assist in crisis administration, even though the expectations of functionality were sometimes distinct.^10,14^

The motivation for individual monitoring of epilepsy encounters obstacles in knowledge and adherence to applications. This was denoted by a study that, through a search on Google Play and the App Store using the keywords “epilepsy” or “seizures”, from May to August 2018, found 22 apps covering different features for self-management of epilepsy. Tracking seizures, maintaining treatment, and medication reminders were also largely overlooked but despite the underutilization of these mechanisms, there was a positive attitude associated with their use as it was pointed out.^16^ Similarly, another study identified 20 smartphone apps with features for self-management of epilepsy, demonstrating a scarcity of these tools in terms of promoting more targeted, individualized, and autonomous self-care, with different performances and distinct aspects of user utilization. This indicates a fragmentation of the required functionalities and the need to engage with more than one application to meet the sought objectives, which tends to reduce adherence.^17^

In existing apps, a higher prevalence of seizure trackers, medication reminders, and individualized action plans within the mobile application were identified, being pointed out as valuable tools along with treatment summaries and appointment calendars.^13^ This demonstrates congruence between the research conducted observationally and reports about existing tools, even though they are fragmented. Additionally, a decrease in the demand for hospital healthcare services was noted through an initiative by a pediatric center to implement these action plans that tend to reduce visits to the emergency department for patients with epilepsy.

The use of apps or devices for managing seizures in people with epilepsy impacts the quality and optimization of treatment. These means enable more personalized care for the individual, with symptom data and seizure history tracked over time, facilitating health education regarding, for example, stress level control, sleep quality, medication administration timing, and consequent assistance in regulating their habits.^10^ These apps also facilitate the work of healthcare professionals, as wearable devices provide real-time and continuous information about the patient’s condition, contributing to faster and individualized treatment with less burden on the healthcare professional compared to traditional methodologies.^12^ Furthermore, the cost of using these apps is relatively affordable, avoiding excessive expenses for both public and private healthcare systems.

The recall of important information was identified as a challenge for both caregivers and patients and online records can provide monitoring with the active involvement of people with epilepsy and their caregivers. One described app featured a seizure diary, medication reminders, self-assessment elements for comorbidities, educational materials, and a personalized dashboard. A prospective study compared health data from clinics with data from mobile apps to determine if there was an improvement in epilepsy self-management with app usage. Through responses to the questionnaire constructed with definitions, safety questions, medication adherence, and crisis management, the authors noted an increase in the possibility of collecting information about each participant’s epilepsy case. This included different types of seizures, sleep deprivation as a triggering factor for the increased frequency of these episodes, and the widespread use of medication reminders. Additionally, there was the potential for suggesting possible neuropsychiatric comorbidities with the concurrent increase in knowledge about the individual’s clinical condition.^6^

On the other hand, there are limitations regarding devices that monitor seizures, as some may be overly sensitive to body movements, leading to false reports. Limited data storage and batteries with low capacity also hinder reliable tracking of information, and there are risks to users regarding the security and privacy of personal data, potentially leading to undesired exposure. The usability of apps by caregivers and patients, especially for external use, is another obstacle to managing these technologies. Additionally, there is sometimes a lack of awareness of the existence of these applications, which tends to generate resistance to the regular use of these mechanisms.^18^ Thus, there are evident difficulties in the acceptability of these technological tools, with indications that, although they provide personalized data, they are limited in their clinical application, as they require further in-depth studies for the pediatric population. As this article is a review, the limitations of the included studies may be reflected in their results. In this context, it is important to note that the available literature for consultation is still limited, the populations in these studies are diverse, and no national studies were located. Furthermore, the population selected for this study was specifically children and adolescents with epilepsy. In the reviewed bibliography, there are commonly identified difficulties in choosing data collection by available apps, with various databases and different methodologies, but generally only using questionnaires and comparisons between clinical reports and elements collected via the app. These technological tools have been developed recently, and it is not possible to guarantee a high level of reliability in seizure monitoring.

In conclusion, the use of apps and mobile devices in managing seizures in children and adolescents with epilepsy has shown promising results by providing continuous and personalized monitoring. This approach represents a significant advancement, offering an alternative technique that facilitates both patient self-management and healthcare professional intervention. Consequently, there is potential for better optimization of seizure control and improved quality of life for patients. It is hoped that future research will further explore the effectiveness of these apps in seizure management, allowing for a more comprehensive understanding of the needs and challenges faced by this population and promoting more effective solutions.
